# Tailoring the specificity of the type C feruloyl esterase FoFaeC from *Fusarium oxysporum* towards methyl sinapate by rational redesign based on small molecule docking simulations

**DOI:** 10.1371/journal.pone.0198127

**Published:** 2018-05-24

**Authors:** Io Antonopoulou, Cameron Hunt, Gabriella Cerullo, Simona Varriale, Alexandra Gerogianni, Vincenza Faraco, Ulrika Rova, Paul Christakopoulos

**Affiliations:** 1 Biochemical Process Engineering, Division of Chemical Engineering, Department of Civil, Environmental and Natural Resources Engineering, Luleå University of Technology, Luleå, Sweden; 2 Department of Chemical Sciences, University of Naples “Federico II”, Naples, Italy; Universität Stuttgart, GERMANY

## Abstract

The type C feruloyl esterase FoFaeC from *Fusarium oxysporum* is a newly discovered enzyme with high potential for use in the hydrolysis of lignocellulosic biomass but it shows low activity towards sinapates. In this work, small molecule docking simulations were employed in order to identify important residues for the binding of the four model methyl esters of hydroxycinnamic acids, methyl ferulate/caffeate/sinapate/*p*-coumarate, to the predicted structure of FoFaeC. Subsequently rational redesign was applied to the enzyme’ active site in order to improve its specificity towards methyl sinapate. A double mutation (F230H/T202V) was considered to provide hydrophobic environment for stabilization of the methoxy substitution on sinapate and a larger binding pocket. Five mutant clones and the wild type were produced in *Pichia pastoris* and biochemically characterized. All clones showed improved activity, substrate affinity, catalytic efficiency and turnover rate compared to the wild type against methyl sinapate, with clone P13 showing a 5-fold improvement in catalytic efficiency. Although the affinity of all mutant clones was improved against the four model substrates, the catalytic efficiency and turnover rate decreased for the substrates containing a hydroxyl substitution.

## Introduction

**Fig 1:**
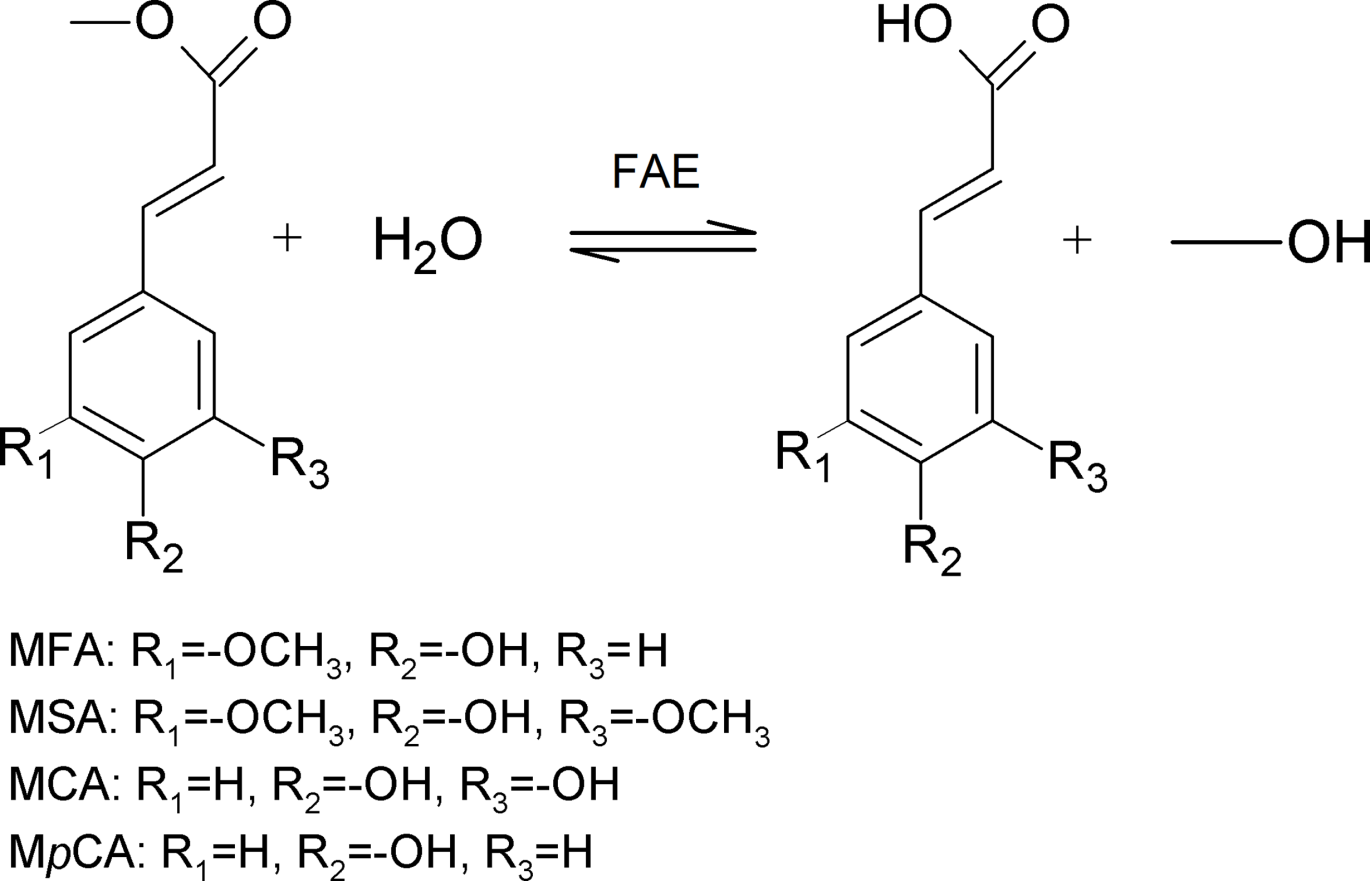
FAE-catalyzed hydrolysis of methyl esters of hydroxycinnamic acids.

Feruloyl esterases (EC 3.1.1.73, FAEs) are a subclass of carbohydrate esterases that are considered a biotechnological key for the degradation of lignocellulosic biomass, catalyzing the hydrolysis of the ester bond between hydroxycinnamic acids, such as ferulic acid (FA), caffeic acid (CA), sinapic acid (SA), *p*-coumaric acid (*p*CA) and sugars found in plant cell walls. Their application as accessory enzymes for hydrolysis as well as for the synthesis of bioactive compounds has been underlined during the past years [1–4]. A widely accepted system for the classification of FAEs is based on their specificity towards the hydrolysis of methyl esters of hydroxycinnamic acids: methyl ferulate (MFA), methyl caffeate (MCA), methyl sinapate (MSA) and methyl *p*-coumarate (M*p*CA) (Fig 1).Type A FAEs show preference on methoxy substituted substrates, such as MFA and MSA, are active on M*p*CA and diferulates but not MCA, while Type B FAEs show preference on hydroxy substituted substrates, M*p*CA and MCA, are active on MFA, but not active against MSA and diferulates. Type C and D have specificity towards all four substrates, but only type D FAEs are active towards diferulates [5–8]. Other classification systems for fungal FAEs have been based on phylogenetic analysis and functionality [9–12].

To date, few type C FAEs have the specificity as it corresponds to the group, such as TsFaeC from *Talaromyces stipitatus* [13], FaeC from *Aspergillus niger* [14] and two FAEs from *Aspergillus terreus* [15]. Others show a profile of type B FAEs with weak or no activity towards MSA, including AnFaeB from *A*. *niger* [6], AoFaeB from *Aspergillus oryzae* [16] and FoFaeC from *Fusarium oxysporum* [17]. Despite their differences in specificity, the aforementioned enzymes were eventually categorized as Type C FAEs due to phylogenetic similarity with tannases [8] [9] [12]. The type C FAE from *Fusarium oxysporum* (FoFaeC) is a newly discovered enzyme with broad pH stability and good synergistic action [17][18]. It belongs to the SF2 subfamily of phylogenetic classification [12] which is closely related to tannases, showing high similarity with AoFaeB from *A*. *oryzae* of known structure [16][19].

Protein engineering is a discipline employing powerful methods, from specific to random, for altering protein function and structure [20]. Engineering of enzyme specificity can be done by rational re-design that is strongly dependent on the detailed understanding of the catalytic mechanisms and determinants of substrate specificity and their use for altering it in a predictable fashion. Several reports exist on rational redesign of different classes of enzymes such as oxidases, esterases, transferases [21–23]. Understanding the mechanisms behind the hydrolytic behavior of FAEs and its relation with the current classification systems is challenging, as FAEs are very diverse enzymes with broad specificity and little unifying sequence. Therefore, applying protein engineering techniques on the modification of the active site of the FAEs could provide insights into their catalytic mechanisms.

**Fig 2:**
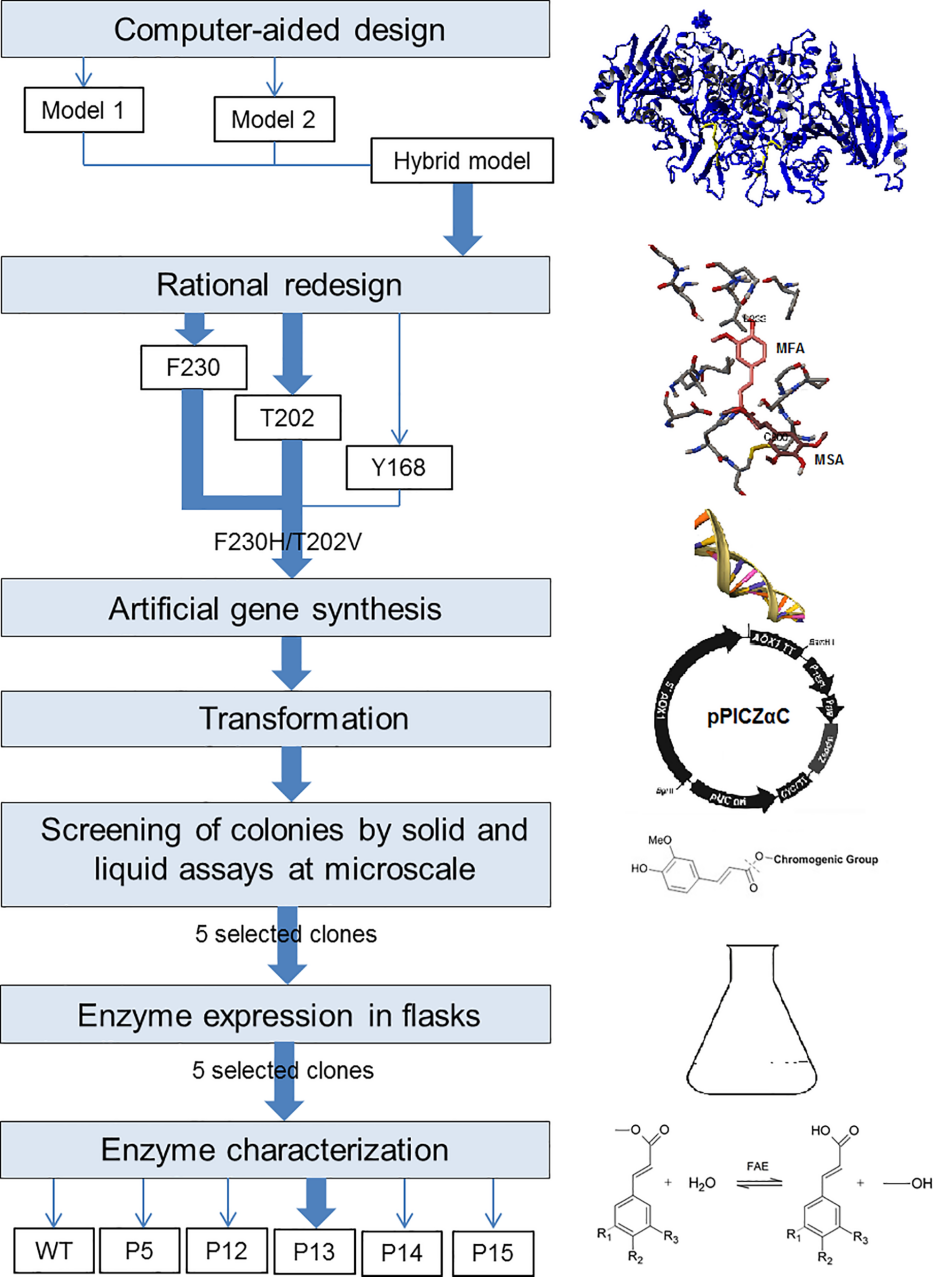
Summary of the followed strategy for the rational redesign of FoFaeC.

In this work, we used protein engineering techniques in order to rationally redesign the active site of the type C FoFaeC from *F*. *oxysporum* aiming to the increase of its activity towards MSA. Via homology modeling, we identified key residues that could possibly inhibit the binding of desired substrate on the enzymes’ active site in a catalytic orientation and suggested substitutions that could benefit the binding via small molecule docking simulations. Subsequently we confirmed the hypothesis by biochemical characterization (Fig 2). To the authors’ knowledge, this is the first report of applying rational protein redesign on a FAE, opening the pathway for understanding the mechanisms behind FAE specificity towards model substrates and tailoring this diverse class of enzymes towards desired bioconversions.

## Materials and methods

### Substrates

MFA and MCA were purchased by Alfa Aesar (Karlsruhe, Germany) while MSA and M*p*CA from Apin Chemicals Ltd. (Abingdon, UK). *p*-Nitrophenyl ferulate (*p*NP-Fe) and 4-nitrocatechol-1-yl ferulate [24] (4NTC-Fe) were provided by Taros Chemicals GmbH & Co. KG (Dortmund, Germany).

### Prediction of FoFaeC structure by homology modeling and small molecule docking simulations

The structure of FoFaeC (Genbank accession number: SCN69328.1) was constructed by homology modeling using YASARA Structure. Possible structural templates for homology modeling were identified by running 3 PSI-BLAST iterations and then searching the Protein Data Bank (PDB) for match (hits with an E-value below the homology modeling cutoff 0.5). Alignment variants of the selected template were developed and refined while a hybrid model was obtained by combining the best part of contributors (developed models). The difference between models and their active site was assessed by calculating the root-mean-square deviation (RMSD) between objects or selected residues, respectively, for each model.

Small molecule docking (SMD) and *in silico* mutational techniques were used to suggest possible mutations that would increase the activity of FoFaeC from *F*. *oxysporum* on MSA. Ligands (MFA, MSA, MCA and M*p*CA) as well as their free acids (FA, SA, CA, *p*CA) were generated using Avogadro [25] and structured optimized using Universal Force Field (UFF). SMD simulations of ligands were performed using Autodock [26] on one monomer the predicted structure of FoFaeC. The exported pdb file was cleaned from water molecules and then converted to a pdbqt involving the addition of polar hydrogen and atom chargers. A grid box was generated around the active site of the enzyme large enough to cover the active site. A standard docking parameter file for each ligand was used for docking. A Lamarckian genetic algorithm was used with 20 runs and a maximum evaluation value of 25000000. Results were visualized using Autodock Tools and evaluated based on the mean binding energy (MBE), number of clusters and number of genetic runs per cluster. Homology models of mutants were generated by swapping residues in YASARA Structure followed by energy minimization.

The volume of the binding pocket was calculated using POVME 2.0 [27]. The center of the inclusion area was specified as the residue furthermost from the catalytic serine that was adjacent to the docked ligand, with the radius being defined as the distance between this residue and the catalytic serine, greater than 1 Å. A grid of points at 1 Å spacing was then generated. Points were then removed being A) lying outside the convex hull of the macromolecule and B) not contiguous with points adjacent to the catalytic serine.

### Strains, vectors and media

*Escherichia coli* TOP10F' strain was used for the amplification of the expression construct pPICZαC/FoFaecMut (Eurofins Genomics, Luxembourg) and the transformants were selected on Low Salt Luria-Bertani medium (1% tryptone; 0.5% yeast extract; 0.5% NaCl pH 7.5) by Zeocin™ resistance (25 μg mL^−1^). The resistant transformants were grown overnight at 37°C under shaking and plasmid DNA was isolated by the Plasmid DNA Extraction Mini Prep Kit (Fisher Molecular Biology, Rome, Italy). The recombinant plasmid pPICZαC/FoFaecMut was linearized with SacI restriction enzyme (NEB, Ipswich, MA, USA) to transform *Pichia pastoris* X-33 (Invitrogen, Carlsbad, CA, USA). The transformation of yeast was performed with 5 μg pure recombinant vector by Electroporation protocol according to the EasySelect™ *Pichia* Expression Kit (Invitrogen, Carlsbad, CA, USA). *P*. *pastoris* transformants were selected on YPDS agar (1% w/v yeast extract; 2% w/v peptone; 2% w/v dextrose; 1 M sorbitol; 2% w/v agar) containing Zeocin™ at final concentration of 100 μg mL^−1^at 28°C. Thirty selected transformants were grown in BMGY and BMMY (1% w/v yeast extract; 2% w/v peptone; 100 mM potassium phosphate, pH 6.0; 1.34% w/v YNB; 4 × 10^−5^% w/v biotin; 1% v/v glycerol or 0.5% v/v methanol) at 28°C.

### Screening of FAE (+) by solid and liquid assays at micro-scale

The thirty colonies were inoculated in 900 μL of BMGY at micro-scale. After incubation at 28°C for 20 h, adequate volume of pre-culture was inoculated in 1 mL of BMMY medium in order to reach optical density (OD600) equal to 1, following incubation for 3 days at 28°C and 700 rpm. Cultures were centrifuged (2500 g, 30 min) and the supernatant from each transformant was transferred to OmniTrays containing 75 μg mL^−1^ of 4NTC-Fe (0.2% v/v stock in DMSO), 50 mM sodium phosphate buffer pH 6.8, 1% w/v agarose and 0.5 mM ammonium iron citrate, necessary for the production of halos, following incubation at 37°C. The supernatant of each transformant found positive in the solid screening assay was analyzed for FAE activity towards *p*NP-Fe according to Mastihuba *et al*. [28], modifying the reaction volume to 1.1 mL and incubation time to 60 min. Activity was also assayed towards MSA at 37°C for 15 min in 100 mM MOPS-NaOH pH 6.0 at a final volume of 1.0 mL. The amount of protein production was detected by the Bradford method (Sigma, Saint-Louis, USA) and the homogeneity was checked by sodium dodecyl sulphate-polyacrylamide gel electrophoresis (SDS-PAGE) stained with Coomassie Blue.

### Production of FAE recombinant clones

Enzyme production was performed in 250 mL flasks with 50 mL of induction medium (BMMY). The cultures were kept in a shaking incubator (180 rpm) at 28°C for 3–5 days with the addition of 0.5% v/v methanol once a day to maintain induction. Cultures were centrifuged (2500 g, 30 min) and the supernatant was collected and 5-fold concentrated. The amount of protein production was detected by the Bradford method (Sigma, Saint-Louis, USA) and the homogeneity was checked by SDS-PAGE stained with Coomassie Blue. The FAE content (% w/w) of each supernatant was estimated by SDS-PAGE and subsequent quantification was done by a densitometric method using JustTLC software (Sweday, Sweden). The wild type and mutant clones expressed FoFaeC in similar levels (average FAE content equal to 89.1±2.2% w/w).

### Characterization of FoFaeC mutant clones and wild type

Characterization experiments took place in a 2 mL Eppendorf thermomixer (Eppendorf, Hamburg, Germany). For the assessment of hydrolytic activity, a stock solution of substrate (50 mM; MFA, MSA, MCA or M*p*CA) was prepared in DMSO. The activity was assayed using 1 mM substrate in 100 mM MOPS-NaOH, pH 6.0 for 15 min at 37°C without agitation varying the enzyme load (0–0.02 mg protein mL^-1^). One unit (1 U) is defined as the amount of enzyme (mg) releasing 1 μmol of hydroxycinnamic acid per minute under the defined conditions. The specific activity was calculated by fitting a linear equation to the acquired data. The effect of substrate concentration on the reaction rate was assessed by incubation of enzyme at varying concentration of substrate (0–2.5 mM) in 100 mM MOPS-NaOH pH 6.0 for 15 min at 37°C. The kinetic constants (*v*_*max*_, *K*_*m*_) were determined by fitting the Michaelis-Menten equation to acquired data using non-linear regression (*p*<0.0001). Reactions were ended by incubating the reaction mixtures at 100°C for 5–10 min. All assays were carried out in duplicate at a final volume of 1 mL and were accompanied by appropriate blanks containing buffer instead of enzyme. There was no hydrolysis observed in the absence of esterase.

### Quantitative analysis of hydroxycinnamates

Analysis was performed by HPLC on a 100–5 C18 Nucleosil column (250 x 4.6 mm) (Macherey Nagel, Düren, Germany). Elution was done with 7:3 v/v acetonitrile: water for 10 min at a flow rate of 0.6 mL min^-1^ and room temperature. Absorbance was measured at 300 nm with a PerkinElmer Flexar UV/Vis detector (Waltham, USA). Retention times for hydrolyzates (FA, CA, SA, *p*CA) and substrates (MFA, MCA, MSA, M*p*CA) were 4.07–4.21 and 5.20–6.21 min, respectively. Calibration curves were prepared using standard solutions of hydroxycinnamates in acetonitrile (0.1–2 mM).

## Results and discussion

### Prediction of FoFaeC structure by homology modeling and comparison with template protein

**Fig 3:**
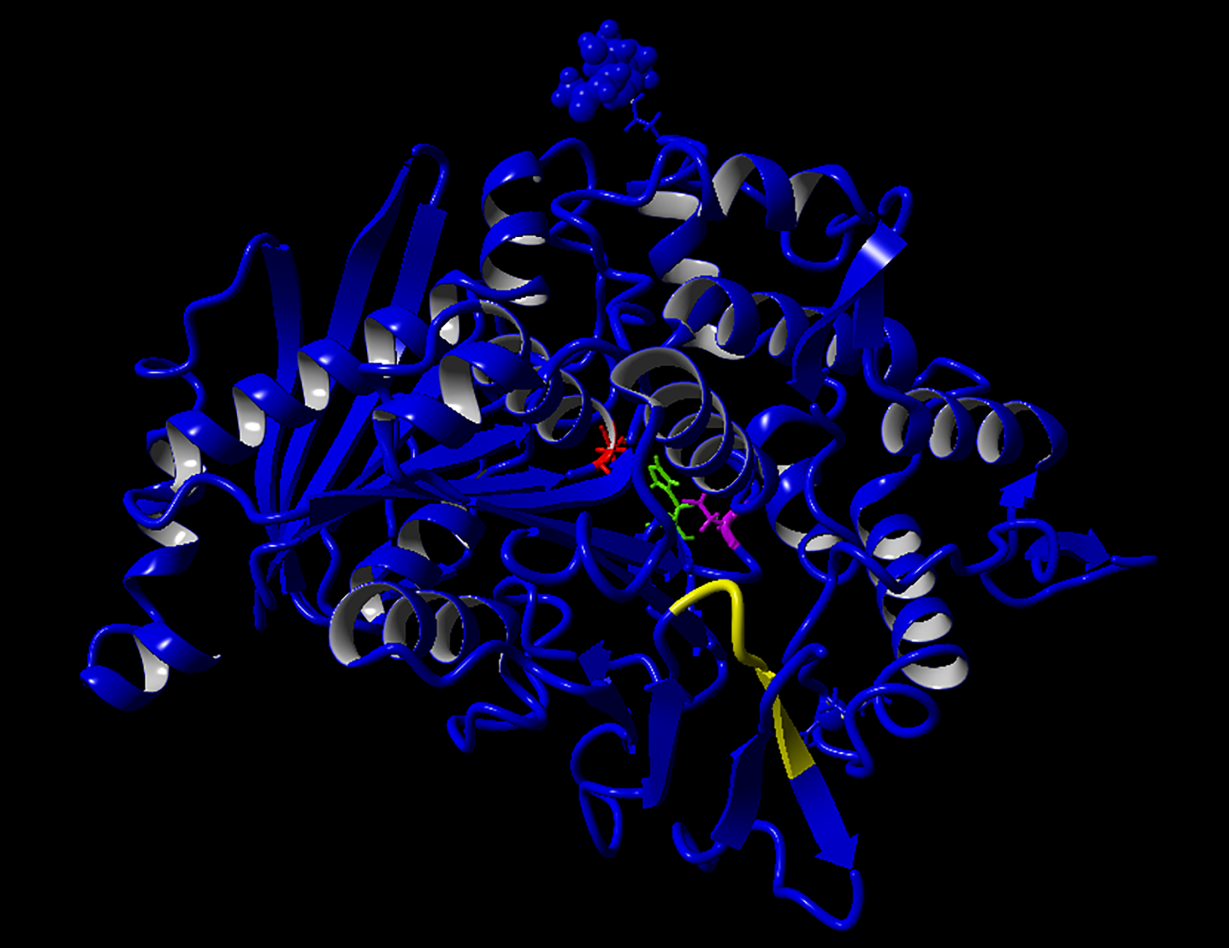
Hybrid model of FoFaeC. Blue: fragment 42–542 from model 1; Yellow: fragment 518–523 from model 2; Red: Ser201; Green: His452; Magenta: Asp412.

The structure of FoFaeC was predicted by homology modeling. Out of thirteen possible identified structural templates, only AoFaeB from *A*. *oryzae* [16] showed significant homology with the target enzyme (identities: 49%; positives: 66%; gaps: 2%; total score 550.50; E-value: 10^−171^), whereas all other templates showed poor homology (Total score 0.00–4.80; E-value: 0.007–0.46). As a next step, two alignment variants were developed based on the determined structure of AoFaeB (PDB: 3WMT) resulting in satisfactory overall quality scores after refinement (-1.122 and 1-.177, respectively). The two models showed no significant overall difference (1.8269 Å RMSD) and no difference at their active sites (0.5366 Å RMSD). Following, the best parts of the models (fragment 42–542 from model 1 and 518–523 from model 2) were combined resulting in a hybrid model, aiming to increase the accuracy (Fig 3). Indeed, the hybrid model exhibited higher quality in terms of overall score (-1.074), dihedrals, 1D and 3D packing comparing to its contributors, as presented in Table 1. Moreover, the difference of the catalytic triad between the hybrid model and contributors was negligible (RMSD equal to 0.1694 Å and 0.4182 Å, respectively). Similarly, superposition of the active site of the hybrid model to model 1 and model 2 (including catalytic triad, disulfide and binding pocket residues) resulted in minimal RMSD equal to 0.3728 and 0.5664 Å respectively. Thus, the hybrid model offering highest quality score was used for SMD simulations and design of mutants. Comparison between the FoFaeC predicted structure (hybrid model) and the determined structure of AoFaeB showed that the binding pocket of FoFaeC is approximately 37.5% smaller while there is 55.6% similarity across the residues identified in the active site of FoFaeC and template (Table 2). Superposition of active site residues resulted in 3.7927 Å RMSD. Finally, superposition of the enzymes’ catalytic triad resulted in negligible RMSD equal to 0.5570 Å. Thus, docking of ligands onto the FoFaeC active site was done according to the orientation of ligands on AoFaeB [16].

**Table 1:** Quality scores for FoFaeC homology models.

Type	Model 1		Model 2		Hybrid model	
	Z-score	Comment	Z-score	Comment	Z-score	Comment
**Dihedrals**	0.238	Optimal	-0.014	Good	0.122	Optimal
**Packing 1D**	-0.897	Good	-0.861	Good	-0.768	Good
**Packing 3D**	-1.734	Satisfactory	-1.734	Satisfactory	-1.704	Satisfactory
**Overall**	-1.122	Satisfactory	-1.122	Satisfactory	-1.074	Satisfactory

Z-score range: Bad (-4 to -3), Optimal (0 to 4)

**Table 2:** Mean binding energy (MBE) and number of docked elements in cluster for the SMD of ligands against FoFaeC wild type.

Ligand	MBE (kcal/mol)	N Clusters
**MFA**	-5.64	5
**MSA**	-^a^	-^a^
**MCA**	-6.20	17
**M*p*CA**	-6.09	19
**FA**	-5.12	4
**SA**	-^a^	-^a^
**CA**	-5.78	19
**M*p*CA**	-5.64	16

Maximum possible elements within an individual cluster are 20.

^a^A more favorable MBE exists in the binding pocket but in a reversed direction

### Docking of methyl esters of hydroxycinnamic acids on the FoFaeC structure

**Fig 4:**
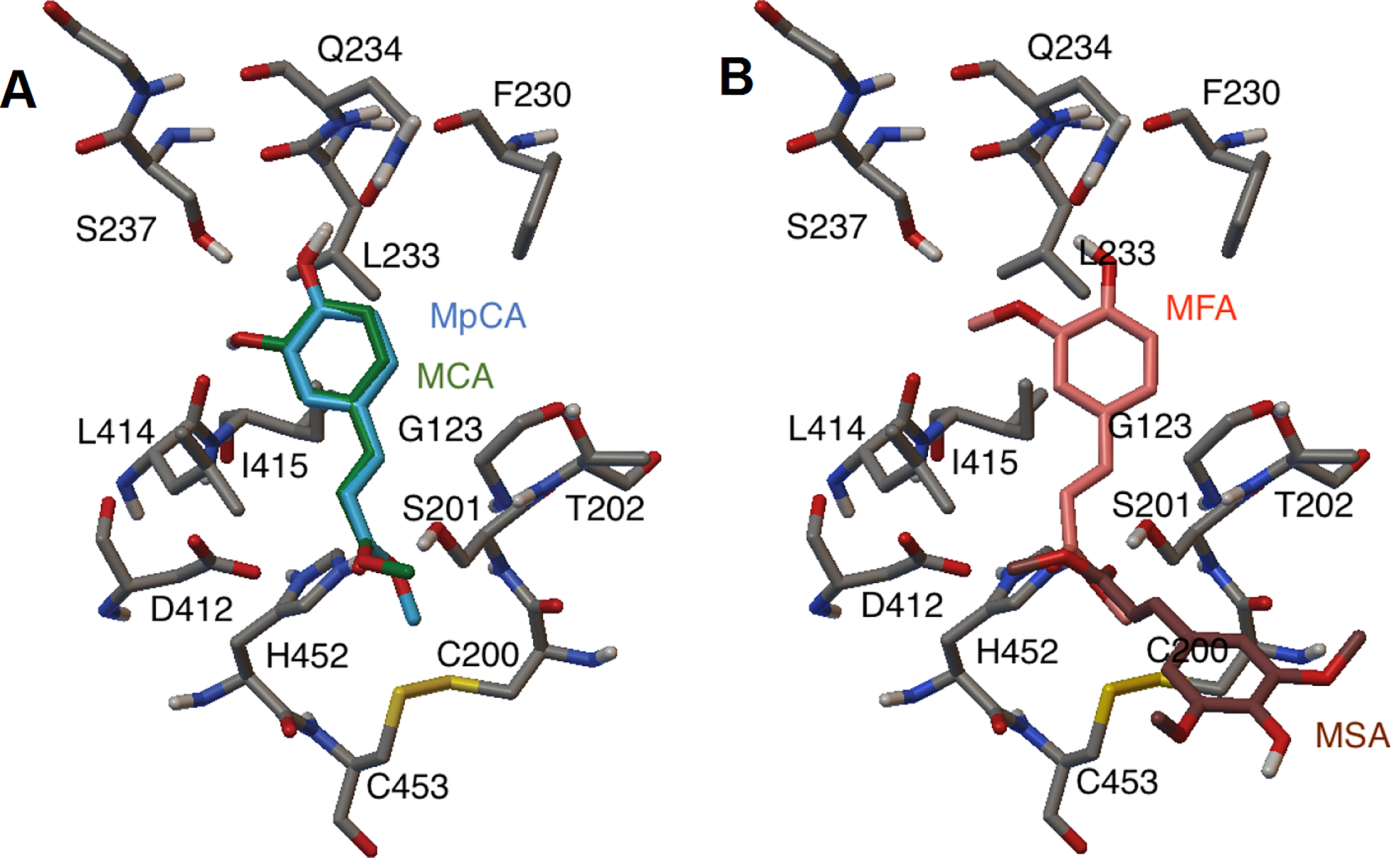
Position of ligands docked into FoFaeC wild type. (A) MCA and M*p*CA (B) MFA and MSA. Phe349 and Tyr351 were removed for clarity.

FoFaeC is a type C FAE that has been shown to have activity against M*p*CA, MCA, MFA and some activity against MSA. Its activity towards MSA is determined significantly lower with a *k*_*cat*_/*K*_*m*_ 50,000 times less than the next closest MCA [17]. Docking of the four model substrates on FoFaeC resulted in a MBE for MCA and M*p*CA equal to -6.09 kcal mol^-1^ and -6.20 kcal mol^-1^, respectively, with high proportion of elements and with the clusters accurately reflecting the high activity of the molecule on these substrates, comparing to MFA (-5.64 kcal mol^-1^) (Table 2). The orientation of the binding of ligands is shown in Fig 4. In the case of hydroxyl substituted esters, the hydroxyl group of the fourth position is hydrogen-bonded to Gln234 and aided by Ser237. MFA, as in the case of AoFaeB, is shifted to the right and downwards but at a lesser degree. The oxygen in the methoxy substitution is stabilized by the serine and the hydrophobic methyl group at residues 414 and 415. The distance between the catalytic Ser201 and the carbonyl carbon is approximately 3.5 Å in all cases. On the other hand, MSA appears to dock in the reversed orientation where the catalytic serine is within a functionally active distance of the carbon carbonyl. This may suggest that the low determined activity of FoFaeC on MSA is not a natural activity orientation of the enzyme on sinapates but an artefact of MSA as the small methyl group allows for this flipped orientation.

### Identification of key residues for MSA activity

As was previously seen, docking of MSA resulted in a reversed orientation than that what was considered for activity as defined by Suzuki *et al*. [16] and the performed SMD for other methyl hydroxycinnamates in this work. Therefore, a synthetic MSA was prepared from the docking of MFA by reflecting the methoxy group perpendicular to the plane of the phenolic ring acquiring the “correct” orientation. Residues were identified to potentially prohibit binding of MSA based on the following assumptions: 1) Side-chains within 1.0 Å of the MSA residue are deemed to produce steric hindrance 2) The methyl group of the methoxy side group requires a hydrophobic environment 3) The oxygen in the methoxy side group should be stabilized by a hydrogen bond.

**Fig 5:**
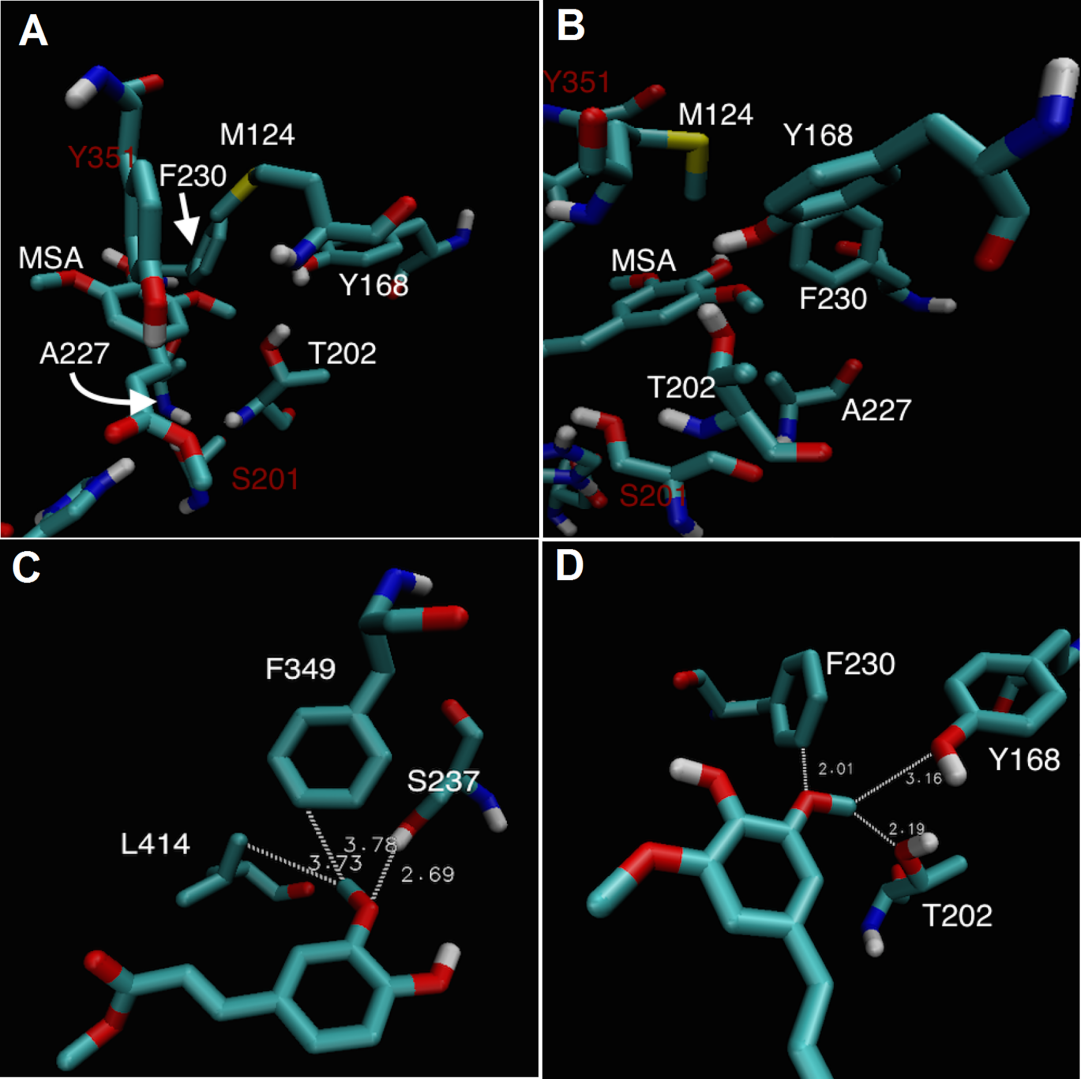
Relevant residues for determining binding of ligand onto FoFaeC wild type. (A) and (B) Binding of MSA representing different angles with Tyr351 and Ser201 residues shown for orientation in red. (C) MFA and (D) MSA binding in reversed orientation showing relevant residues involved in side group recognition.

The following residues were deemed to be relevant as they existed within a 10.0 Å radius of MSA side group: Met124 placed above the binding pocket, Thr202 placed in the hydrophilic side chain and in close contact with the methyl group of the methoxy side chain of MSA, Phe230 which is hydrophobic and placed at the back of pocket near the oxygen of the methoxy side group of MSA, Tyr168 placed far right of the pocket providing small hydrophobic environment and Ala227 below the pocket (Fig 5A and 5B).

**Fig 6:**
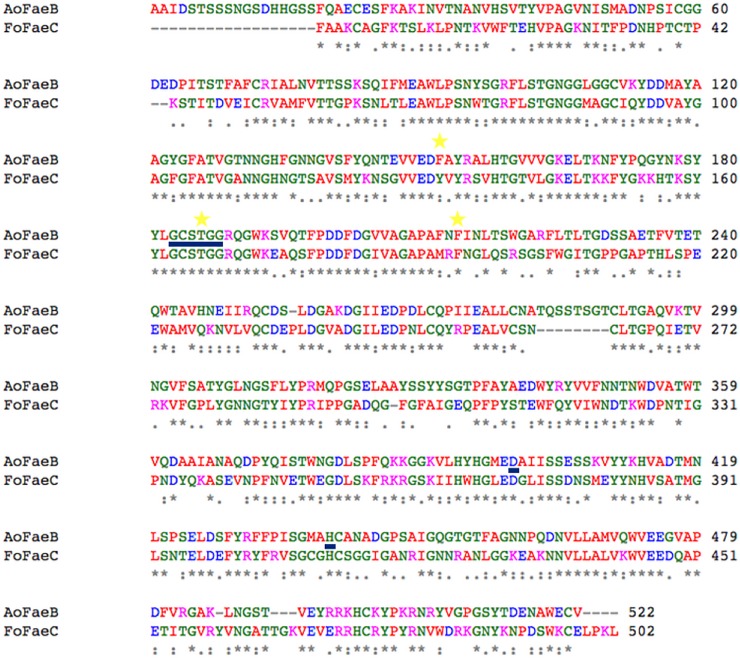
Sequence alignment between AoFaeB and FoFaeC. The catalytic residues are underlined in blue and the potential MSA interacting residues underlined with a star.

Furthermore, analysis of the original methoxy side group for the binding of MFA highlights three important aspects. The distance between the methyl group and the nearest hydrophobic residues is 3.73 Å and 3.78 Å (a leucine and a phenylalanine, respectively) (Fig 5C). Additionally, the oxygen is stabilized by a hydrogen bond to a serine residue at a distance of 2.69 Å. Analysis of methoxy side group for the off-side reversed binding of MSA reveals that there is a distance of 2.19 Å between the methyl group and polar threonine, 3.16 Å to the polar tyrosine and 2.01 Å to the phenylalanine from the oxygen with this being non-polar/hydrophobic (Fig 5D). Of the three residues highlighted previously, two of them are also found in AoFaeB with the third tyrosine being substituted by a phenylalanine (Fig 6). As AoFaeB does not have activity on MSA and the low activity of FoFaeC could be considered an artefact, consensus residues are likely a good candidate for substitution. Thr202 does occur as part of the nucleophilic elbow GCSTGG but is not one of the consensus residues.

### Prediction of mutations for increasing activity towards MSA

According to the previous observations, possible substitutions, based on the need of a polar group to support oxygen and a non-polar group to support the methyl group, are: Phe230 to a bulky polar residue such as histidine (F230H), serine (F230S) or tyrosine (F230Y) in order to increase distance, Thr202 to a hydrophobic valine (T202V) or alanine (T202A) and Tyr168 to a large hydrophobic residue such as phenylalanine (Y168F). Homology models of the six individual mutants were generated in order to identify the effect of mutation on the distance to the methoxy side group of MSA. In particular, F230H and F230Y increased the distance between the polar group on residue 230 and the oxygen to 2.98 Å and 3.33 Å, respectively. This is within the expected range for moderate hydrogen bonding (2.5–3.2 Å) [29]. The mutation F230S increased the distance to 5.35 Å far beyond the needed for hydrogen bonding. The threonine mutations T202V and T202A increased the distance between the methyl group and the now hydrophobic side group to 3.28 Å and 3.48 Å, respectively. The single mutation Y168H increased the distance between the methyl group and the hydrophobic side chain of residue 168 to 4.31 Å. While a true, optimal size for a hydrophobic pocket is hard to estimate, these distances are far greater than the 1 Å requirements and within the same range for the methoxy side group of FA moiety, as described by Suzuki *et al*. [16] for AoFaeB from *A*. *oryzae* and by Hermoso *et al*. [30] for AnFaeA from *A*. *niger*.

The effect of the single mutation on the distance was used to direct the creation of double or triple mutants. Two triple mutants where selected as the Y168F residue was deemed necessary along with the T202A mutation allowing more room within the binding pocket. Additionally, an alanine substitution was thought to provide better stability to the nucleophilic elbow. The two triple mutants F230H/T202A/Y168F and F230Y/T202A/Y168F were similarly modeled and all eight structures, five single mutants excluding F230S, the two double mutants and the wild type were used as receptors for SMD with both MFA and MSA as ligands. A grid box was created according to the larger binding pocket of mutants, thus binding of MSA to wild type was achieved and MBE of MFA was differentiated.

**Fig 7:**
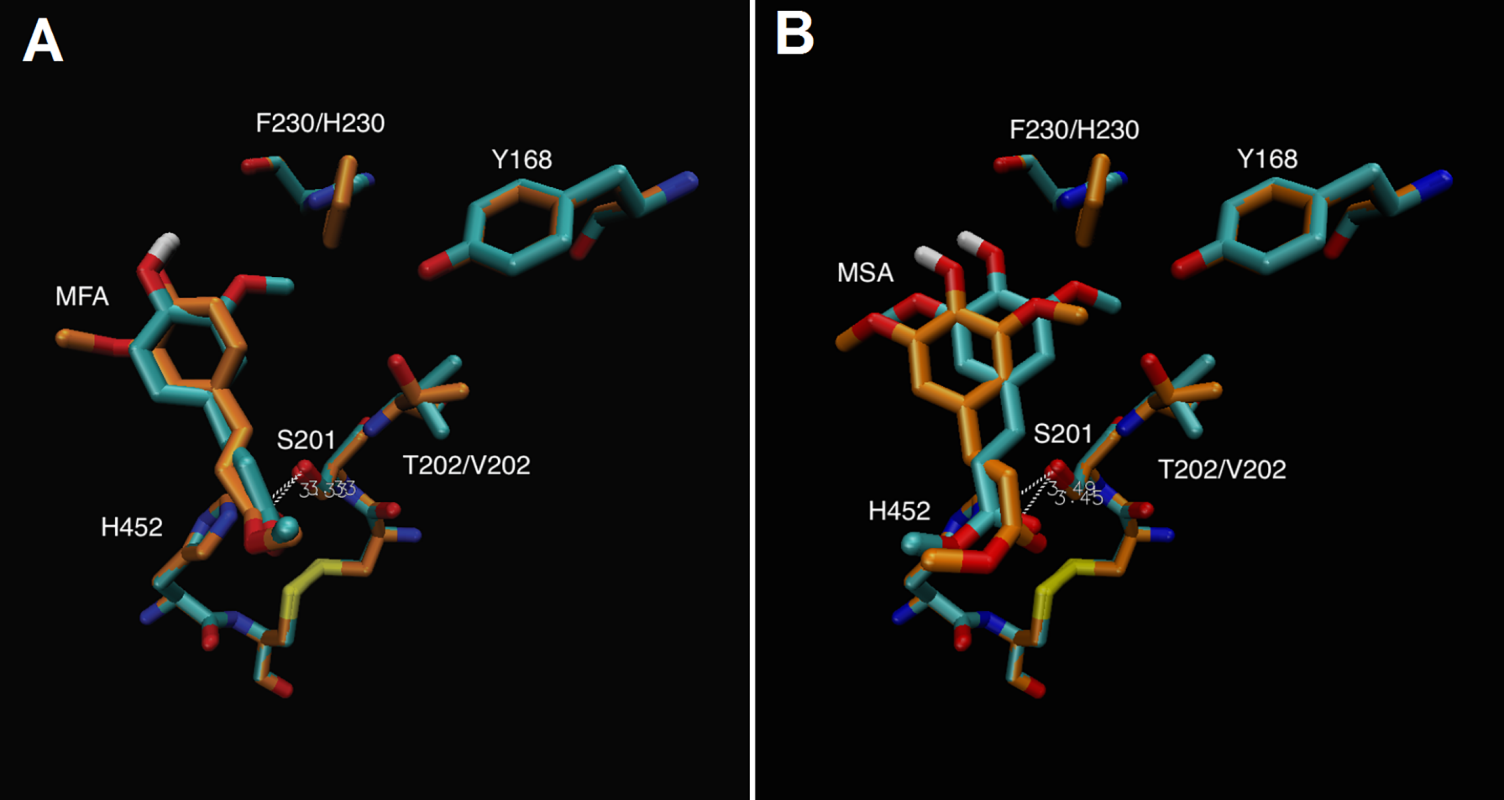
FoFaeC double mutant F230H/T202V (blue) compared with wild type (orange). (A) Docked MFA and (B) Docked MSA.

The results were assessed in terms of MBE, orientation of binding and number of clusters represented in binding RMSD (Table 3). While the highest increase in MBE was only 0.4 kcal mol^-1^, the increase in the number of elements within the cluster is far more significant indicating that of the 20 genetic algorithm runs, 10 resulted in the desired orientation. The Y168F mutation appears to have little effect on the docking of MSA and thus could be omitted. Single mutation F230H appears to create a large number of positive clusters while the F230H triple mutant was the most successful. T202V was more suspenseful than T202A therefore one further double mutant F230H/T202V and a triple mutant F230H/T202V/Y168H were generated. As presented in Table 3, SMD revealed that the T202V mutation on the triple mutant is more effective than the T202A decreasing the distance to the methyl group from the hydrophobic side chain and causing less torsion on the MSA. It also shows that the Y168F mutation is unnecessary and provided no additional stability to the hydrophobic nature of the pocket. From this observation the double mutant F230H/202V was recommended to increase activity of MSA. Fig 7 shows the docking of MFA and MSA against the selected mutant and the wild type. Both mutations combined open up the right side of the pocket allowing the fitting of the methoxy group and subsequently the “correct” and catalytic binding of MSA. The distance between the catalytic serine and the carbonyl carbon is around 3.3 Å. The double mutant F230H/T202V increases the MBE to -5.50 kcal mol^-1^ and increases the number of runs in that cluster.

**Table 3:** Mean binding energy (MBE) and number of docked elements in cluster for SMD of MFA and MSA to FoFaeC mutants and wild type.

Enzyme	MFA		MSA	
	MBE (kcal/mol)	N Clusters	MBE (kcal/mol)	N Clusters
**Wild type**	-5.28	16	-5.03	2
**F230H**	-5.39	20	-5.30	13
**F230Y**	-5.35	17	-5.17	3
**T202A**	-5.27	12	-5.33	9
**T202V**	-5.28	16	-5.43	10
**Y168F**	-5.29	17	-4.99	2
**F230H/T202A/Y168F**	-5.40	18	-5.38	15
**F230Y/T202A/Y168F**	-5.27	14	-5.29	6
**F230H/T202V**	-5.28	19	-5.50	17
**F230H/T202V/Y168H**	-5.40	18	-5.51	13

The maximum possible elements within an individual cluster are 20.

### Recombinant expression in *P*. *pastoris* X33 and screening of transformants in solid and liquid media

**Fig 8:**
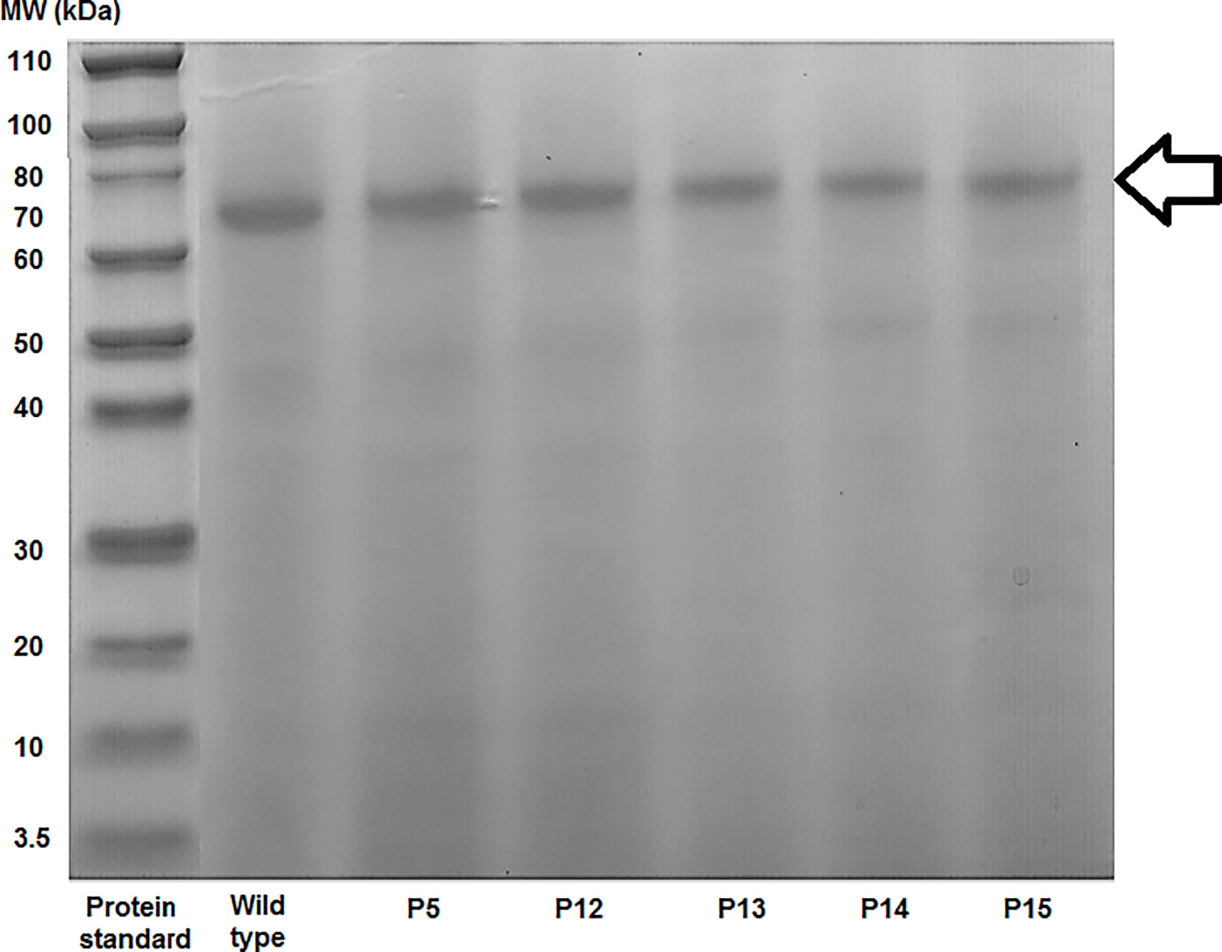
SDS-PAGE of mutant clones and wild type. 2.5–4.1 μg (20 μL) of concentrated supernatant was loaded in each well.

A synthetic gene was designed incorporating the most promising mutation (F230H/T202V) and was recombined in *P*. *pastoris* X33. Thirty colonies from *P*. *pastoris* X33 transformants were grown in micro-scale. After three days of incubation at 28°C, culture supernatants were recovered and spotted on solid media containing 4NTC-Fe. Twenty out of thirty clones were active showing activity halos (data not shown). Subsequently, the supernatants from fifteen transformants were analyzed for FAE activity in liquid medium towards *p*NP-Fe. Both assays were performed using wild-type strain as negative control and *P*. *pastoris* recombinant producing FoFaeC wild type as the positive sample. From these analyses, less than fifteen clones out of thirty analyzed were active (data not shown). Based on these results, five transformants (P5, P12, P13, P14 and P15) were chosen to scale-up FAE production in 250 mL-flasks. The homogeneity of each culture was checked by SDS-PAGE while the FAE content was higher than 89% for each transformant, as determined by densitometric analysis (Fig 8).

The cultures were incubated at 28°C for 3–5 days and after biomass removal, the supernatant was analyzed for the FAE activity against *p*NP-Fe and MSA. The preliminary screening showed that FoFaeC wild type had activity towards *p*NP-Fe but no activity was detected towards MSA. However, the transformants showed activity for MSA while the activity towards *p*NP-Fe was more than halved. Moreover, no activity was detected in any case for non-transformed *P*. *pastoris* strain.

### Characterization of mutant clones and wild type

**Fig 9:**
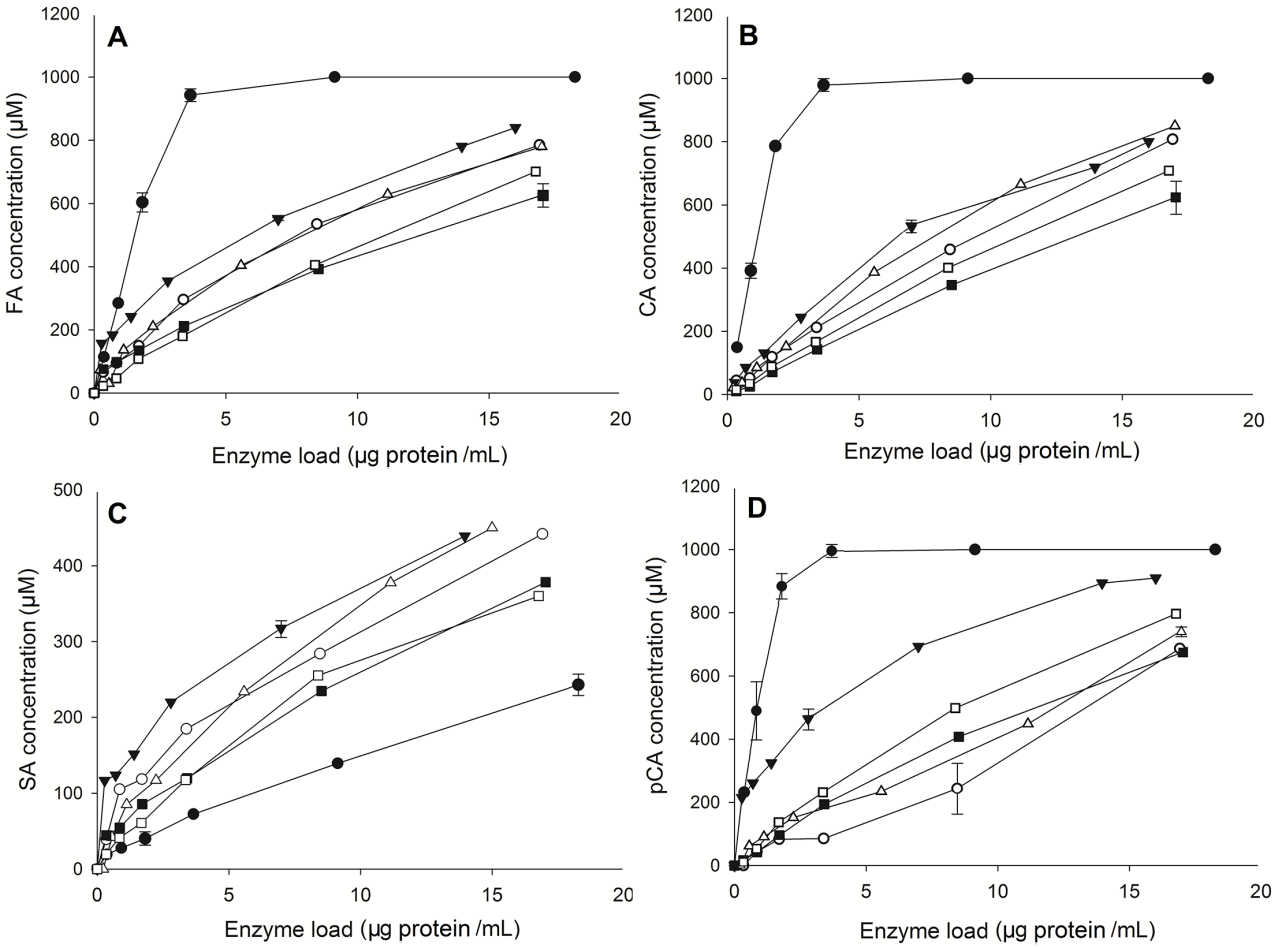
Effect of enzyme load on the release of hydroxycinnamic acid during hydrolysis of methyl esters. (A) MFA (B) MCA (C) MSA (D) M*p*CA. Black circle: wild type; white circle: P5; black triangle: P12; white triangle: P13 black square: P14; white square: P15.

The five FoFaeC (P5, P12, P13, P14, P15) mutant clones carrying the double mutation F230H/T202V and the wild type were 5-fold concentrated and further characterized for their activity towards the four methyl esters of hydroxycinnamic acid (MFA, MSA, M*p*CA and MCA) using varying enzyme load (0–0.02 mg protein mL^-1^). The wild type of FoFaeC showed highest activity in descending order against M*p*CA> MCA> MFA> MSA. In accordance with previous report [17], some activity towards MSA could be detected; however it was approximately 20 times lower than MFA. Validating our hypothesis, all mutant clones showed improved activity towards MSA compared with the wild type (Table 4). Mutant P13 showed highest specific activity towards MSA, approximately 5 times higher than the wild type, followed by mutant P15 and P12. Interestingly, the activity of mutant clones towards the other substrates was dramatically decreased but remained in the same order of magnitude with MSA. More specifically, the activity towards MFA was 5-fold decreased while towards hydroxyl substituted substrates, such as M*p*CA and MCA, was 10-fold decreased. The difference of the specific activity between different clones of the mutation F230H/T202V is owed to different levels of total protein expression for each clone. At the same time, the levels of FoFaeC expression differ for each clone as estimated by SDS-PAGE, probably due to multiple gene insertion events at a single locus in a cell occurring spontaneously with a low but detectable frequency in the *P*. *Pastoris* expression system. The effect of enzyme load on the release of hydroxycinnamic acids is presented in Fig 9.

**Table 4:** Relative specific activities of FoFaeC mutant clones and wild type against methyl esters of hydroxycinnamic acids.

Enzyme	Relative specific activity (% U mg^-1^ total protein)			
	MFA	MSA	MCA	M*p*CA
**Wild type**	54.2 (2.9)	2.8 (0.1)	85.1 (0.5)	100 (4.7)
**P5**	13.4 (0.9)	5.7 (1.4)	9.8 (0.3)	15.8 (0.5)
**P12**	17.9 (2.4)	7.1 (2.7)	15.6 (0.7)	15.0 (1.9)
**P13**	12.1 (0.6)	14.7 (0.5)	12.2 (0.2)	14.1 (0.6)
**P14**	7.9 (0.6)	4.7 (0.3)	7.4 (0.1)	8.2 (0.3)
**P15**	8.6 (0.3)	9.3 (0.6)	8.6 (0.2)	12.1 (0.5)

Numbers in parentheses represent the standard error from curve fitting

**Fig 10:**
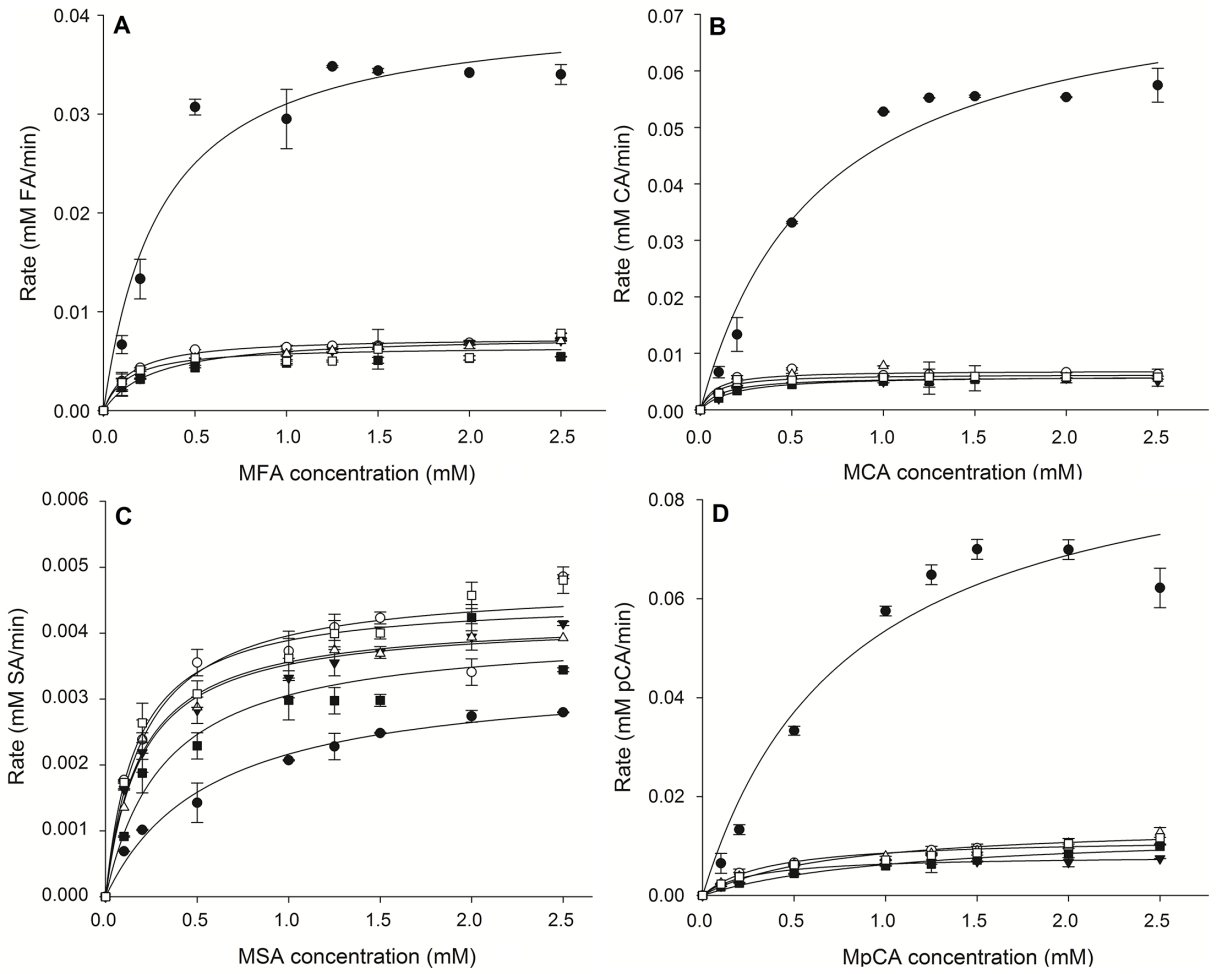
Effect of substrate concentration on the reaction rate during hydrolysis. (A) MFA (B) MCA (C) MSA (D) M*p*CA. Black circle: wild type; white circle: P5; black triangle: P12; white triangle: P13 black square: P14; white square: P15.

Results on the effect of substrate concentration on the hydrolysis rate revealed that the FoFaeC wild type in this study has higher affinity (lower *K*_*m*_) towards methoxy substituted esters (MFA> MSA> MCA> M*p*CA) and higher turnover rate (higher *k*_*cat*_) against hydroxyl substituted esters (M*p*CA> MCA> MFA> MSA) (Table 5). Generally, all mutant clones showed improved affinity against all esters compared to the wild type but, and in particular when MSA was used, the reaction rate was 1.5-fold increased. The catalytic efficiency (*k*_*cat*_/*K*_*m*_) of mutant P13 towards sinapate was 5-fold improved comparing to that of the wild type while the affinity was 2-fold increased. The effect of substrate concentration on the reaction rate is shown in Fig 10. An explanation on the higher affinity of mutant clones towards the hydroxy substituted esters could be that the addition of histidine expands the binding pocket offering binding of substrates in the correct conformation (lower *K*_*m*_). This is also predicted by the increased number of elements within a cluster for docking of MFA on the active site of mutant compared to the wild type (Table 3). However, the lower reaction rates (approximately 10-fold decrease) could be attributed to the small hydrophobic environment introduced by valine, which could be opposing the hydroxyl group of substitution ester and resulting in a not so catalytically favorable orientation of the carbonyl carbon.

**Table 5:** Kinetic constants of FoFaeC mutant clones and wild type.

Enzyme	*K*_*m*_ (mM)	*v*_*max*_ (μmol L^-1^ min^-1^)	*k*_*cat*_ (min^-1^)	*k*_*cat*_/*K*_*m*_
**MFA**				
**Wild type**	0.331 (0.102)	41.0 (3.6)	1392 (122)	4466 (1431)
**P5**	0.141 (0.015)	7.4 (0.1)	271 (3.7)	1925 (206)
**P12**	0.276 (0.036)	7.6 (0.2)	338 (8.9)	1221 (162)
**P13**	0.155 (0.021)	7.0 (0.2)	389 (11.1)	2519 (348)
**P14**	0.153 (0.009)	5.7 (0.0)	208 (0.36)	1359 (80.0)
**P15**	0.133 (0.065)	6.5 (0.6)	240 (22.2)	1813 (901)
**MSA**				
**Wild type**	0.424 (0.063)	3.0 (0.1)	102 (3.4)	241 (36.7)
**P5**	0.201 (0.036)	4.9 (0.2)	180 (7.3)	894 (164)
**P12**	0.194 (0.030)	4.2 (0.1)	187 (4.5)	963 (151)
**P13**	0.189 (0.022)	4.2 (0.1)	234 (5.6)	1236 (147)
**P14**	0.245 (0.045)	3.6 (0.2)	131 (7.3)	535 (103)
**P15**	0.200 (0.047)	4.8 (0.2)	178 (7.4)	887 (212)
**MCA**				
**Wild type**	0.649 (0.183)	77.4 (7.4)	2628 (251)	4051 (1206)
**P5**	0.075 (0.035)	6.9 (0.5)	253 (18.3)	3882 (1833)
**P12**	0.118 (0.025)	5.9 (0.2)	262 (8.9)	2222 (477)
**P13**	0.218 (0.027)	9.3 (0.4)	517 (22.2)	2379 (312)
**P14**	0.177 (0.016)	6.1 (0.1)	222 (3.6)	1258 (156)
**P15**	0.074 (0.021)	6.1 (0.2)	226 (7.4)	3069 (877)
**M*p*CA**				
**Wild type**	0.799 (0.312)	96.4 (14.1)	3273 (478)	4094 (1707)
**P5**	0.352 (0.036)	11.6 (0.3)	425 (10.9)	1209 (128)
**P12**	0.275 (0.030)	8.1 (0.2)	360 (8.9)	1310 (152)
**P13**	0.719 (0.225)	14.6 (1.6)	812 (88.9)	1130 (375)
**P14**	1.122 (0.326)	13.3 (1.7)	484 (61.9)	431 (137)
**P15**	0.678 (0.202)	13.5 (1.4)	499 (51.8)	736 (232)

Numbers in parentheses represent the standard error from curve fitting

## Conclusions

The rational redesign of the active site of type C FoFaeC provided an insight into the hydrolytic mechanisms of this enzyme and opens the way for a new approach on the exploitation of FAEs for use in novel bio catalytic processes by tailoring their specificity according to the desired reaction.
